# Integrated Management of Multiple Sclerosis Spasticity and Associated Symptoms Using the Spasticity-Plus Syndrome Concept: Results of a Structured Specialists' Discussion Using the Workmat^®^ Methodology

**DOI:** 10.3389/fneur.2021.722801

**Published:** 2021-09-27

**Authors:** Oscar Fernandez, Lucienne Costa-Frossard, Maria Luisa Martínez-Ginés, Paloma Montero, Jose María Prieto-González, Lluís Ramió-Torrentà

**Affiliations:** ^1^Department of Pharmacology, Biomedical Research Institute of Malaga, University of Málaga, Málaga, Spain; ^2^Department of Neurology, Hospital Universitario Ramón y Cajal, Madrid, Spain; ^3^Department of Neurology, Hospital Gregorio Marañón, Madrid, Spain; ^4^Department of Neurology, Hospital Clínico San Carlos, Madrid, Spain; ^5^Department of Neurology, Complejo Hospitalario Universitario de Santiago, Santiago de Compostela, Spain; ^6^Department of Neurology, Hospital Universitari de Girona Doctor Josep Trueta, Girona, Spain; ^7^Neuroimmunology and Multiple Sclerosis Unit, Girona Biomedical Research Institute (IDIBGI), Girona, Spain; ^8^Medical Sciences Department, University of Girona, Girona, Spain

**Keywords:** multiple sclerosis, spasticity, cannabidiol (CBD), symptom management, symptomatic treatment, tetrahydrocannabinol (THC), syndrome, quality of life

## Abstract

**Background:** Multiple sclerosis (MS) treatment has radically improved over the last years; however, MS symptom management is still challenging. The novel Spasticity-Plus syndrome was conceptualized to frame several spasticity-related symptoms that can be addressed together with broad-spectrum medication, such as certain cannabinoid-based drugs. The aim of this project was to gain insight into Spanish neurologists' clinical experience on MS spasticity and associated symptoms, and to assess the acknowledgment and applicability of the Spasticity-Plus syndrome concept in patients with MS.

**Methods:** Ten online meetings were conducted using the Workmat^®^ methodology to allow structured discussions. Fifty-five Spanish neurologists, experts in MS management, completed and discussed a set of predefined exercises comprising MS symptom assessment and its management in clinical practice, MS symptoms clustering in clinical practice, and their perception of the Spasticity-Plus syndrome concept. This document presents the quantitative and qualitative results of these discussions.

**Results:** The specialists considered that polytherapy is a common concern in MS and that simplifying the management of MS spasticity and associated manifestations could be useful. They generally agreed that MS spasticity should be diagnosed before moderate or severe forms appear. According to the neurologists' clinical experience, symptoms commonly associated with MS spasticity included spasms/cramps (100% of the specialists), pain (85%), bladder dysfunction (62%), bowel dysfunction (42%), sleep disorders (42%), and sexual dysfunction (40%). The multiple correspondence analysis revealed two main symptom clusters: spasticity-spasms/cramps-pain, and ataxia-instability-vertigo. Twelve out of 16 symptoms (75%) were scored >7 in a 0–10 QoL impact scale by the specialists, representing a moderate–high impact. The MS specialists considered that pain, spasticity, spasms/cramps, bladder dysfunction, and depression should be a treatment priority given their frequency and chance of therapeutic success. The neurologists agreed on the usefulness of the new Spasticity-Plus syndrome concept to manage spasticity and associated symptoms together, and their experience with treatments targeting the cannabinoid system was satisfactory.

**Conclusions:** The applicability of the new concept of Spasticity-Plus in MS clinical practice seems possible and may lead to an integrated management of several MS symptoms, thus reducing the treatment burden of disease symptoms.

## Introduction

Multiple sclerosis (MS) is a chronic, immune-mediated disease of the central nervous system and one of the most common causes of neurological disability in young adults globally (1). In 2020, there were approximately 2.8 million people with MS worldwide (2), with prevalence rates varying among countries, being around 80–180 cases per 100,000 in Spain (3).

The etiology of MS is multifactorial (4), resulting in a wide heterogeneity of clinical presentations, disease severity and response to treatments (5). There are three main pharmacological treatment groups in MS: disease-modifying therapies (DMT), treatments targeting relapses, and those addressing symptomatology (6). MS therapy has radically improved over the last years, particularly DMTs targeting relapsing–remitting MS (7, 8). These new therapeutic options have increased life expectancy (9). The management of MS is also challenged due to the wide range of potential symptoms, including gait/mobility impairment (related to spasticity, ataxia, tremor, and progressive disability), bladder, bowel and sexual dysfunction, pain, fatigue, cognitive impairment, psychological and psychiatric conditions, visual and brainstem symptoms, sleep disorders, and paroxysmal symptoms (10). Since MS symptoms substantially impact the quality of life (QoL) and daily activities of patients, effective management of MS symptomatology is key.

As most MS symptomatology treatments target individual symptoms, managing them requires a multidisciplinary approach and often results in polytherapy (11). Polytherapy increases the risk of side effects and drug interactions and can exacerbate other MS symptoms (12–14). Therefore, simplifying MS symptom management can have important benefits for patients. An approach to achieve this goal is to identify symptoms that share a common underlying pathological mechanism and/or that may benefit from being treated with a single therapy. Based on this rationale, we recently defined the Spasticity-Plus syndrome concept, which encompasses a group of symptoms associated with MS spasticity with a common or close pathophysiology and/or responding to the same treatment (11). These symptoms include spasticity, spasms/cramps, pain, bladder dysfunction, fatigue, and sleep disorders (11).

Spasticity is one of the most common MS symptoms, with a prevalence over 80% in patients with MS (15, 16). This symptom is characterized by muscle stiffness that can lead to pain, spasms, and reduced mobility, deeply impairing QoL (17). Moreover, the increased muscle tone can trigger or worsen non-mobility MS symptoms, such as fatigue, sleep disorders, and bladder dysfunction (17), several of them mediated in the brainstem or close areas (11). The cannabinoid receptors CB1 and CB2 are distributed across the central nervous system and are highly expressed in the brainstem (18). Nabiximols, an oromucosal spray containing tetrahydrocannabinol (THC) and cannabidiol (CBD) together with other cannabinoids and components (Sativex^®^), interact with CB1 and CB2 receptors to modulate the endocannabinoid system (19). The improvement in treatment-resistant spasticity to the most commonly used drugs (baclofen, tizanidine, diazepam, or combinations) with nabiximols has been demonstrated in several randomized clinical trials (19–22), and this improvement was interestingly associated with improvement of other symptoms that could be therefore considered as associated symptoms to spasticity such as pain, sleep disorders, and bladder dysfunction in MS patients (19–21, 23).

Taking together all this information, the authors previously hypothesized (11) that framing these symptoms within a syndrome, the Spasticity-Plus syndrome, and treating them with therapies targeting the cannabinoid system may benefit both spasticity and its associated symptoms.

Given the novelty of the Spasticity-Plus syndrome concept, we conducted a series of structured discussions among a panel of 55 neurologists specialists in MS across Spain to gain insight into their clinical experience related to MS spasticity and associated symptoms, to assess the acknowledgment and applicability of the Spasticity-Plus syndrome concept in MS management, and to better organize clinical data collection in the future.

## Materials and Methods

A panel of 55 neurologists, experts in the management of MS in Spain, completed and discussed a set of predefined exercises following the Workmat^®^ methodology (24, 25). Workmat^®^ is a structured discussion methodology based on the promotion of debate and sharing of clinical experience among a group of experts (24, 25). Different specific exercises were designed, tested, and validated by a Scientific Committee composed of six neurologists with recognized expertise in MS spasticity management and who previously participated in the Spasticity-Plus syndrome concept definition (11).

Before their allocated discussion meeting, the MS specialists read the paper conceptualizing the Spasticity-Plus syndrome and completed four online exercises covering different aspects of MS symptomatology, with particular focus on spasticity. Ten online discussion meetings, each one of them with five to six neurologists coordinated by a member of the Scientific Committee, were conducted between September and October 2020, lasting around 2 h each. The discussions consisted of a structured discussion of the results of previous exercises presented in a slide format in which the specialists could provide additional insights into each topic.

The exercises were divided into three main sections: (i) MS symptom assessment and management in clinical practice, covering aspects such as symptom communication by the patient, diagnosis, polypharmacy or patient's education; (ii) MS symptoms clustering in clinical practice, focusing on the identification of potential clusters of MS symptoms, the estimated impact of each symptom on patient's QoL and ranking of symptoms by treatment priority; and iii) discussion of the Spasticity-Plus syndrome concept.

The present document shows the results of the in-depth discussions among the participating specialists and the compiled results of the exercises performed.

### Data Analysis

Fifty-seven neurologists specializing in MS, coming from MS units that follow-up at least several hundred people with MS, distributed all over Spain, were invited to participate; 55 of them (96.5%) accepted the invitation. Discussions and exercises results were analyzed both quantitatively and qualitatively.

To assess the experience of the specialists with MS symptoms management, in the first of the three sections, 13 statements were discussed, covering aspects such as symptom assessment and follow-up, symptom communication, symptom awareness by the patient, frequency of follow-up visits, suitability of checklists to detect spasticity-associated symptoms, side effects of polypharmacy, treatment simplification, and assessment of spasticity and associated symptoms. Results were described qualitatively after conducting a content analysis of the meeting recordings.

The second section, MS symptoms clustering, comprised three exercises based on 16 hallmark MS symptoms (pain, spasticity, spasms/cramps, bladder dysfunction, depression, fatigue, sleep disorders, sexual dysfunction, bowel dysfunction, instability, ataxia, cognitive impairment, vertigo, vision loss, sensory alterations, and dysarthria) (11). In the first exercise, the experts clustered the symptoms by joint onset (if they occurred concomitantly) and by common pathophysiology using different colors. Based on the data provided by the neurologists in this exercise, a hierarchical cluster analysis was carried out for each classification, joint onset and common pathophysiology, respectively. Cluster analysis is a multivariate technique whose aim is to classify objects formed by groups (clusters) that are as much homogeneous as possible within themselves and heterogeneous among themselves. We arranged the data provided by participants in a matrix of n individuals and p variables and calculated the matrix of distances between variables for each clustering exercise using the squared Euclidian distance, which is the most appropriate for the type of variables in the study. As the first step, a matrix of distances between variables was calculated. We used Ward's (26) minimum variance criterion that minimizes the total within-cluster variance for the classification of clusters. This method tends to form compact clusters of equal size and shape and is very few sensitive to extreme values. Afterwards, dendrograms were constructed to graphically display the results of each clustering exercise. This method of clustering of variables is similar to a principal components analysis (PCA) but, while the PCA requires strong assumptions (linearity, normality, etc.), cluster analysis is less restrictive in its assumptions (it does not require linearity, symmetry, etc.) and admits different methods to estimate the distance matrix depending on the characteristics of the data. SPSS, version 22 was used in this analysis. The second exercise of this section consisted of rating the impact of each symptom on the QoL of patients using a 10-point scale (0 = no impact, 10 = greatest impact). Finally, as a third exercise in this section, the experts ordered the symptoms from lowest (1) to highest (16) by treatment priority, considering both their frequency and the possibility of managing them effectively.

The third section of the project was focused on the assessment of their degree of agreement with the Spasticity-Plus syndrome concept, by rating on a 10-point scale (0 = totally disagree, 10 = totally agree) 10 statements, four related to symptoms management and six to the Spasticity-Plus syndrome concept. The results were presented as a spider map.

## Results

### Section 1. MS Symptom Assessment in Clinical Practice

The specialists acknowledged the complexity of MS clinical expression, with patients often suffering from a wide range of symptoms. In their experience, the number of symptoms assessed during hospital visits is highly dependent on the time available per visit and on whether it is a specialized MS consultation or not. In a routine clinical visit, neurologists usually do not address all the potential MS symptoms but only those previously reported by the patient. A detailed assessment of symptoms through an in-depth structured anamnesis is more likely when the hospital has monographic MS units.

The specialists concluded that MS patient education is crucial to detect symptoms early and to improve disease management. However, some experts considered that providing too much information may increase a patient's concerns and induce symptoms suggestion. Key aspects of patient communication are the proactive role of the patient and clear and efficient communication, tailoring the information to the patient's disability status according to the Expanded Disability Status Scale (EDSS) and the presence of other related symptoms.

#### MS Spasticity

The panel reported that they only inform patients in advance about the possibility of suffering spasticity and associated symptoms in later stages of MS, when their occurrence is more frequent. According to their experience, patients usually receive information on spasticity and associated symptoms once spasticity is diagnosed. Patients frequently are not aware of spasticity-associated symptoms, and the proactive communication of these manifestations to their physician is often unclear or imprecise, typically expressing their impact on daily activities. Moreover, patients may mislead general symptoms with MS relapses or flare-ups.

Although the specialists generally agreed that spasticity should be diagnosed before moderate or severe forms appear, many neurologists recognized that a later-stage diagnosis is frequent. Some experts considered that in the real world, control visit intervals are too wide to detect mild manifestations of spasticity. Few of them reported using specific scales for spasticity, such as Ashworth or 0–10 Numeric Rating Scale (NRS). In contrast, the frequency of visits (less than a visit every 6 months) to follow up spasticity once diagnosed was generally considered adequate in most centers, although the experts also noted that symptomatic treatments should be followed more regularly to confirm their effectiveness and patient's tolerability. Most experts considered that the availability of specific questionnaires or checklists addressing spasticity and associated symptoms would improve diagnosis and may contribute to a more efficient patient exploration.

The specialists highlighted the importance of initiating MS spasticity treatment early, preferably through non-pharmacological approaches such as physiotherapy to avoid or reduce polypharmacy. They generally agreed that both pharmacological and non-pharmacological approaches should be considered when there is an impact on QoL. The importance of tailoring the pharmacological treatment to the circumstances of each patient was also acknowledged, since some patients may benefit from some levels of spasticity to perform certain daily activities such as walking or standing.

It was generally agreed that polypharmacy is common among MS patients with spasticity and associated symptoms, although the experts recognized their effort to minimize it. They also pointed out that other healthcare professionals may negatively interfere in the treatment of spasticity and associated symptoms by adding drugs for other conditions, for instance. The experts agreed that managing each symptom independently increases the risk of side-effects and drug interactions, although the latter is less frequent. It was concluded that simplifying the management of clinical manifestations associated with spasticity could reduce the side effects of polypharmacy in MS patients.

### Section 2. MS Symptom Clustering in Clinical Practice

#### MS Symptom Clusters

##### Symptoms Associated With Spasticity

According to the panelists, the symptoms most commonly associated with spasticity onset (concomitant occurrence) were spasms/cramps (100% of experts) and related pain (85% of them). Bladder dysfunction, bowel dysfunction, sleep disorders and sexual dysfunction were grouped with spasticity onset by 62%, 42%, 42%, and 40% of the experts, respectively. For most experts (98%), spasms/cramps and spasticity share underlying pathophysiology, followed by pain (67%) and bladder dysfunction (56%).

##### Multiple Correspondence Analysis

**Figure 1:**
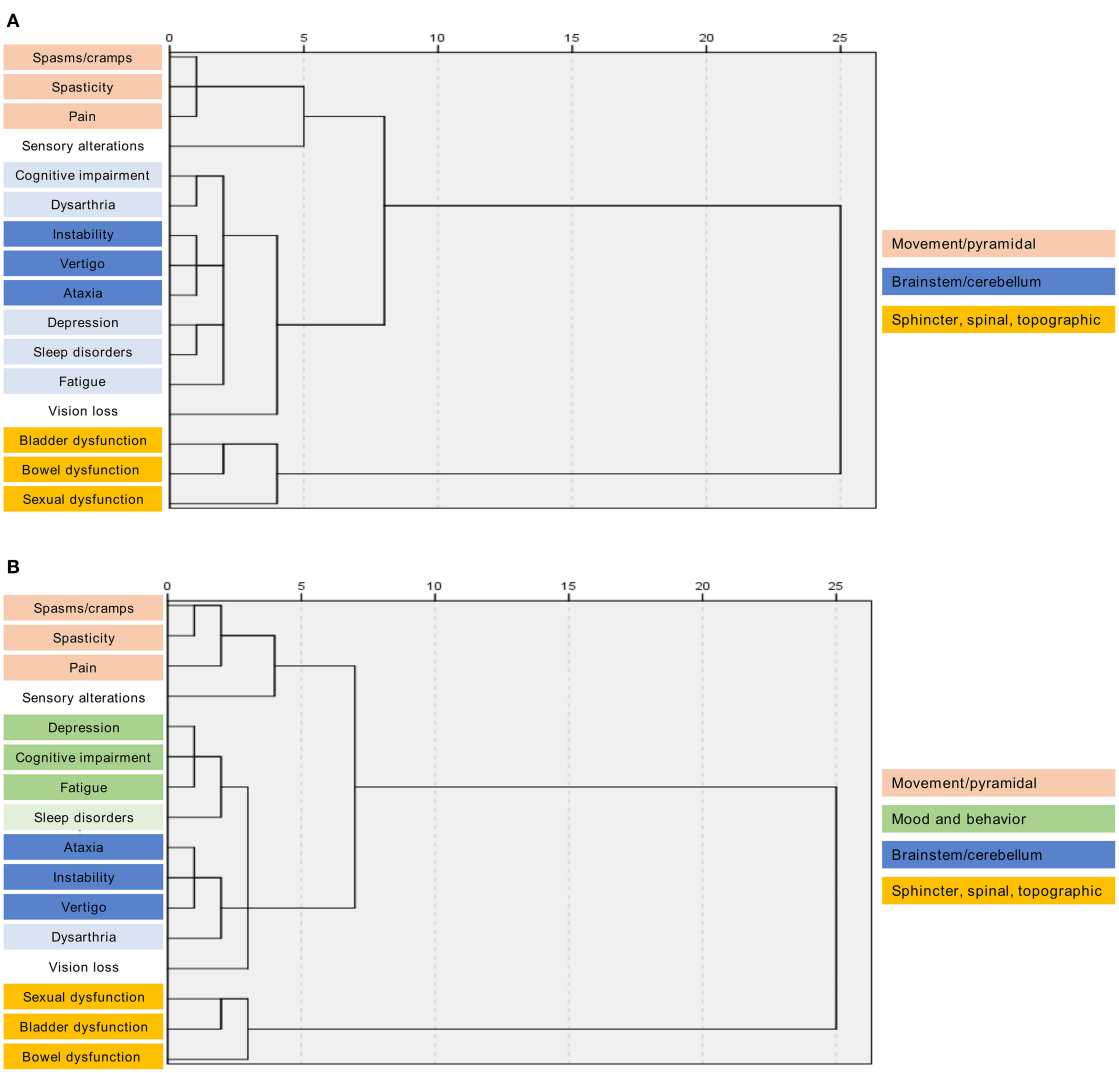
Hierarchical clustering of multiple sclerosis symptoms by joint onset or common pathophysiology. Dendrograms show symptom clusters by **(A)** joint onset or **(B)** common pathophysiology. The x-axis represents the distance between nodes. Symptoms were clustered using Ward's method.

We used multiple correspondence analysis to identify MS symptom clusters with joint onset (Figure 1A) or common pathophysiology (Figure 1B). Two major clusters were detected in both classifications: i) spasticity-spasms/cramps-pain, and ii) ataxia-instability-vertigo (Figures 1A,B). Genitourinary symptoms were often clustered together (Figures 1A,B) and could be related to spasticity in case of spinal cord MS lesions, according to the majority of the panelists. During the discussions, the specialists emphasized the importance of sexual dysfunction and highlighted the difference between masculine and feminine dysfunction. While masculine dysfunction is frequently associated with bladder control problems, feminine dysfunction is possibly more associated with psychological causes.

Depression, cognitive impairment, and fatigue were clustered by common pathophysiology (Figure 1B). However, mood and behavior manifestations, although common in many patients, are non-specific symptoms and can have mixed origins or even be unrelated.

Sensory alterations and vision loss were not clearly associated with any pathophysiology or joint onset cluster (Figures 1A,B). The experts highlighted that sensory alterations might be secondary to MS lesions in sensory structures (brainstem, cerebellum, or posterior cord) and related to neuropathic pain and hypoesthesia. Vision impairment is generally related to optic neuritis with vision loss or visual acuity and, in some cases, to nystagmus, altered accommodation, or diplopia.

According to the experts, the lack of a clear correlation between sleep disorders and any cluster could be explained considering that sleep disorders appear as a consequence of a number of MS-related symptoms: spasms, pain, and nycturia may challenge sleep quality, whereas mood and behavior symptoms may trigger insomnia. It was concluded that clustering symptoms within the Spasticity-Plus syndrome concept may become clearer as MS evolves.

#### MS Symptoms Impact on QoL

**Figure 2:**
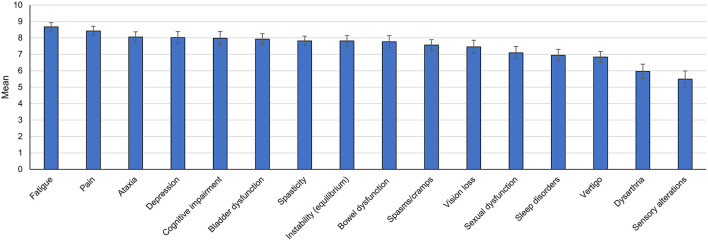
Impact of multiple sclerosis symptoms on quality of life according to the panel opinion. The bar graph shows mean scores (95% confidence interval). Each multiple sclerosis symptom was rated on a 10-point scale (0 = no impact, 10 = greatest impact).

Twelve out of 16 MS symptoms (75%) were scored >7 on a 0–10 scale, representing a moderate–high impact on the patients' QoL. The symptoms considered as more severely affecting QoL were fatigue (score 8.67), pain (8.42), and ataxia (8.05). The mean score of spasticity was 7.82 points, while sensory alterations showed the lowest score (5.49) (Figure 2).

During the discussions of these results, the specialists considered that the influence of certain symptoms on QoL perception may depend on specific circumstances of the patient, such as occupation. Moreover, the impact of some disabling symptoms on QoL may be lower than expected in patients with a higher EDSS due to the accommodation of expectations associated with worse health status.

Within the MS symptoms considered less severely impairing QoL (lower scores), the specialists noted that sexual dysfunction may negatively impact younger patients. The low score given to vision loss compared to other MS symptoms could be explained by its reduced incidence among MS patients. The experts also indicated that sensory alterations such as paresthesia are more easily coped with than other MS symptoms.

#### MS Symptoms Treatment Priority

The specialists considered that pain (mean ranking position 3.57), spasticity (4.07), spasms/cramps (4.67), bladder dysfunction (5.13), and depression (5.48) are the symptoms with highest priority, on a range from 1 to 16. In contrast, vertigo (mean ranking position, 11.09), vision loss (12.13), sensory alterations (12.44), and dysarthria (13.46) scored the lowest in treatment priority (Table 1).

**Table 1:** Treatment prioritization of multiple sclerosis symptoms.

**Symptoms**	**Mean**	**95% CI**
**Pain**	3.57	4.50;2.65
**Spasticity**	4.07	4.72;3.43
**Spasms/cramps**	4.67	5.43;3.90
**Bladder dysfunction**	5.13	5.88;4.38
**Depression**	5.48	6.47;4.49
**Fatigue**	6.78	7.88;5.68
**Sleep disorders**	7.63	8.55;6.71
**Sexual dysfunction**	8.78	9.55;8.01
**Bowel dysfunction**	8.98	9.99;7.97
**Instability (equilibrium)**	10.50	11.49;9.51
**Ataxia**	10.59	11.60;9.58
**Cognitive impairment**	10.69	11.61;9.76
**Vertigo**	11.09	12.02;10.16
**Vision loss**	12.13	13.21;11.05
**Sensory alterations**	12.44	13.49;11.40
**Dysarthria**	13.46	14.26;12.66

*Data are expressed as mean scores (95% confidence interval) of each multiple sclerosis symptom ordered by treatment priority from 1 to 16 (lower scores indicate higher priority)*.

Pain was considered the highest priority because it is a frequent symptom among MS patients, and several therapeutic options are available for its management. Spasticity and spasms/cramps are also frequent and interconnected symptoms with effective therapeutic interventions. Similarly, bladder dysfunction and depression are common in MS patients, which can be reasonably managed with existing therapies. The specialists stressed that, despite fatigue being common in MS and severely impacting patients' QoL, specific and efficacious therapies to manage this symptom are scarce and frequently improve with the management of other symptoms. Likewise, efficacious treatment options to manage cognitive impairment or dysarthria are limited.

The specialists emphasized that several symptoms can be treated with a single therapy, either due to the action of the drug itself or due to the cause-consequence interrelation of different symptoms. Therefore, involved neurologists frequently prioritize the management of those symptoms with a higher chance of therapeutic success, or treatment of which can allow the concurrent improvement of more symptoms.

### Section 3. Spasticity-Plus Syndrome Concept Agreement

**Figure 3:**
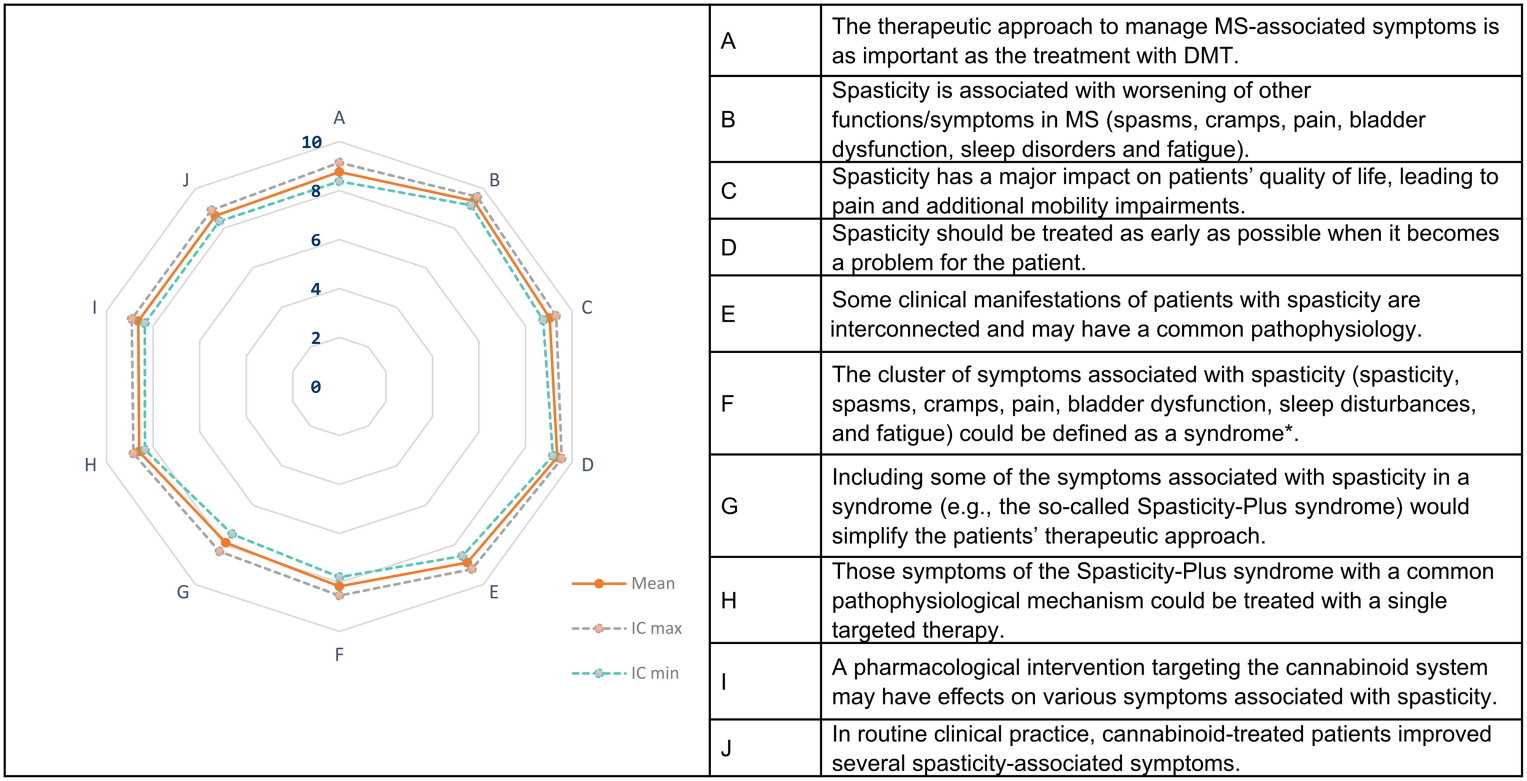
Spasticity-Plus syndrome concept. The spider map shows the mean scores (95% confidence interval) for each statement scored on a 10- point scale (0 = totally disagree, 10 = totally agree). MS, multiple sclerosis; DMT, disease-modifying treatment. *Syndrome is defined as a combination of signs and/or symptoms that form a distinct clinical entity indicative of a disease or disorder.

A high level of agreement (mean score 8.73 out of 10) was observed on all of the items regarding the concept of Spasticity-Plus syndrome. The panelists agreed on the clinical relevance of spasticity and associated symptoms, also because of their association with the exacerbation of other MS symptoms (mean score, 9.36/10) and the loss of QoL (mean score, 9.04/10) (Figure 3, Table 2).

**Table 2:** Spasticity-Plus syndrome concept.

***N* = 55**	**Symptoms**				**Treatment approach**					
	**A**	**B**	**C**	**D**	**E**	**F**	**G**	**H**	**I**	**J**
**Mean**	8.76	9.36	9.04	9.36	8.89	8.16	7.89	8.60	8.64	8.62
**SD**	1.41	0.80	1.04	0.70	1.23	1.40	1.62	0.91	1.04	1.03
**95% CI**	0.38	0.22	0.28	0.19	0.33	0.38	0.44	0.25	0.28	0.28
**95% CI upper limit**	9.15	9.58	9.32	9.55	9.22	8.54	8.33	8.85	8.92	8.90
**95% CI lower limit**	8.38	9.15	8.76	9.17	8.56	7.79	7.45	8.35	8.35	8.34
**Min**	4	7	6	7	5	4	3	7	7	6
**Max**	10	10	10	10	10	10	10	10	10	10
**Median**	9	10	9	9	9	8	8	9	9	9
**P25**	8.00	9.00	8.00	9.00	8.00	8.00	7.00	8.00	8.00	8.00
**P75**	10.00	10.00	10.00	10.00	10.00	9.00	9.00	9.00	9.50	9.00

A, The therapeutic approach to manage MS-associated symptoms is as important as the treatment with DMT; B, Spasticity is associated with worsening of other functions/symptoms in MS (spasms, cramps, pain, bladder dysfunction, sleep disorders and fatigue); C, Spasticity has a major impact on patients' quality of life, leading to pain and additional mobility impairments; D, Spasticity should be treated as early as possible when it becomes a problem for the patient; E, Some clinical manifestations of patients with spasticity are interconnected and may have a common pathophysiology; F, The cluster of symptoms associated with spasticity (spasticity, spasms, cramps, pain, bladder dysfunction, sleep disturbances, and fatigue) could be defined as a syndrome^*^; G, Including some of the symptoms associated with spasticity in a syndrome (e.g., the so-called Spasticity-Plus syndrome) would simplify the patients' therapeutic approach; H, Those symptoms of the Spasticity-Plus syndrome with a common pathophysiological mechanism could be treated with a single targeted therapy; I, A pharmacological intervention targeting the cannabinoid system may have effects on various symptoms associated with spasticity; J, In routine clinical practice, cannabinoid-treated patients improved several spasticity-associated symptoms. CI, confidence interval; SD, standard deviation. *Syndrome is defined as a combination of signs and/or symptoms that form a distinct clinical entity indicative of a disease or disorder.

A wide consensus was reached on the importance of grouping several interconnected symptoms such as spasticity, spasms/cramps, pain, bladder dysfunction, fatigue, and sleep disorders within the Spasticity-Plus syndrome for comprehensive and integrated symptom management. The specialists also widely agreed on the definition of *syndrome* as a cluster of symptoms associated with spasticity (mean score 8.16/10) and the possibility of simplifying the therapeutic approach by grouping these symptoms within a syndrome (mean score 7.89/10) (Figure 3). Although during the discussions some specialists questioned the suitability of the term syndrome, most of them agreed on the usefulness of optimizing the treatment of spasticity-associated symptoms through this integrative approach.

It was concluded that there might be a common underlying pathophysiology between spasticity and associated symptoms (mean score, 8.89/10) that could explain why a pharmacological intervention targeting the cannabinoid system may have effects on several symptoms associated with MS spasticity (mean score, 8.64/10). In routine clinical practice, an approved pharmacological intervention targeting the cannabinoid system (nabiximols) can improve different spasticity-associated symptoms (mean score, 8.62/10), with specialists reporting satisfactory, and even very satisfactory, results on several manifestations, particularly in spastic bladder, spasms and pain (some also in sleep disorders) (Figure 3).

A number of aspects to consider regarding Nabiximols treatment were identified during the discussions. The importance of a progressive dose adjustment and of a close patient follow-up to minimize tolerability issues was pointed out. The specialists also considered important to educate patients on Nabiximols as a medication approved by the medicines agencies to avoid misinterpretations and relationship with direct herbal cannabis/marijuana, leading to patient reluctance or wrong perceptions about the presence of adverse events such as dizziness or somnolence. Another issue highlighted by the specialists was the taste of the product, which may reduce treatment adherence. Some neurologists also claimed administrative access difficulties, with strict restrictions on the use of Nabiximols on a wide range of MS patients by hospital pharmacies.

## Discussion

This study evaluated the acknowledgment and applicability of the new concept of Spasticity-Plus syndrome and may pave the way for future investigations assessing its applicability in clinical practice. Despite the wide range of available MS therapies, disease management continues to be a challenge for patients and healthcare providers (4). In particular, due to the heterogeneity of symptoms, symptomatic therapy may lead to complex treatments with multiple drugs, which increases the risk of adverse events and drug interactions and may exacerbate other symptoms (11, 27). Among these symptoms, spasticity is very disabling and highly prevalent in patients with MS (16, 28). Spasticity can trigger or worsen other MS symptoms, such as fatigue, sleep disorders, and bladder dysfunction, requiring a multidisciplinary approach for their management (17). One of the conclusions reached during the meetings was the importance of an active screening of symptoms and of an effective communication with the patient to allow early recognition and diagnosis of spasticity symptoms. To this end, the information provided to patients should be tailored considering the patient's disability status and the presence of related symptoms evaluated through the EDSS and specific symptom tests. The same applies to the communication of spasticity and associated symptoms, as specialists suggested informing patients about the possibility of experiencing these symptoms only in advanced stages of the disease when the likelihood of their occurrence is higher.

The specialists agreed that early diagnosis of MS spasticity is crucial, as previously recommended (29). However, they also acknowledged that spasticity is often diagnosed too late, given the low frequency of clinical consultations, one of the key barriers to early diagnosis. The experts also recognized that specific questionnaires or checklists for spasticity diagnosis could be useful and efficient, particularly in settings under time constraints.

The challenge that polypharmacy poses for patients and healthcare professionals was highlighted. In this regard, a recent systematic review showed the correlation of polypharmacy with comorbidities, increased disability, cognitive impairment, hospitalizations, relapses, and QoL impairment in patients with MS (13). Therefore, simplifying the drug management of spasticity and associated symptoms is an opportunity to reduce the side-effects related to polypharmacy.

The symptoms that the experts most frequently associated with spasticity onset were spasms/cramps and pain, followed by bladder dysfunction, bowel dysfunction, sleep disorders, and sexual dysfunction. The identification of this group of symptoms with a common onset or pathophysiology reinforces the concept of Spasticity-Plus syndrome, which included spasticity, spasms/cramps, pain, bladder dysfunction, sleep disorders, fatigue, and/or tremor (11) and is in agreement with previous studies (17, 30). Therefore, only few differences are observed in the group of symptoms previously defined in the Spasticity-Plus syndrome concept (11) and the set obtained after the discussion sessions. This may be explained because the latter is based on neurologists' experience and opinion, while the Spasticity-Plus syndrome was conceptualized based on the improvements observed in several symptoms in clinical trials assessing the efficacy of Nabiximols on spasticity. Correspondence analysis of expert data identified two main MS symptom clusters by joint onset or common pathophysiology: (1) spasticity-spasms/cramps-pain, and (2) ataxia-instability-vertigo. One of the conclusions of the discussions is that clustering symptoms within the Spasticity-Plus syndrome concept may become clearer as MS evolves.

The panel considered that those symptoms compromising patient independence (ataxia, pain and cognitive impairment) and general status (fatigue and depression) more deeply impact the QoL of MS patients. It is important to highlight that the Spasticity-Plus syndrome comprised several symptoms (fatigue, pain, or bladder dysfunction) that were ranked among the top positions. The impact of symptoms such as fatigue, cognitive impairment, and sexual and bladder dysfunction on the QoL of patients with MS has been previously reported (31–35).

Several symptoms included in the Spasticity-Plus syndrome (pain, spasticity, spasms/cramps, and bladder dysfunction) were identified as top treatment priorities. It is important to note that limiting symptoms such as fatigue were not prioritized because unfortunately, scarce specific and efficacious therapies to manage this symptom are available. The low prioritization of symptoms such as fatigue is in line with the findings of a large German MS registry analysis showing that sexual dysfunction, dysphagia, cognitive dysfunction, and fatigue were treated to a lesser extent (36).

The importance of grouping several symptoms with common or close pathophysiology and/or responding to a single therapy revealed high agreement. In this context, the novel concept of Spasticity-Plus syndrome may help optimize the pharmacological management of symptoms, with important benefits for patients and healthcare providers. Implementing the new concept of Spasticity-Plus in clinical practice may lead to an integrated management of MS symptoms, which can reduce disease and treatment burden. The experts acknowledged that having in mind the idea of a syndrome may foster the screening of potentially related symptoms that can be concurrently managed, thus reducing polypharmacy. In general, the experience with Nabiximols treatment reported by the participating specialists in the improvement of spasticity and associated symptoms was satisfactory and should be taken further with future studies. Future research should evaluate to which extent the different symptoms included in the Spasticity-Plus syndrome improve upon Nabiximols treatment in prospective and retrospective studies.

Previous evidence supports the development of the Spasticity-Plus concept. Initial studies indicated that fatigue, depression, and pain encompass a symptom cluster that negatively correlates with physical activity (37) and reduced QoL (34, 35). The authors of these studies already pointed out a potential biological link between these symptoms and highlighted the importance of developing interventions to manage them concurrently (34, 35). A cross-sectional study identified seven clusters among 24 MS symptoms in relapsing–remitting MS, several of them tightly associated with patient-reported outcomes: the cluster of pain, muscle spasms, and stiffness was related to physical QoL, depression influenced mental QoL, and cognitive difficulty was associated with work impairment (38). The analysis of cross-sectional data of patients with MS identified that fatigue, anxiety and sleep disturbance mediate the effects of chronic pain on depression, suggesting that treatments addressing sleep, fatigue, and anxiety may improve chronic pain and depressive symptoms (39). The importance of comprehensive management of symptom clusters in MS was also emphasized in a review (40). In a recent pilot study, the MS symptom cluster of pain, fatigue, and depression was successfully targeted with a single-treatment approach based on transcranial direct current stimulation (41). The present study is in line with these data and identifies other potential symptom clusters that can be concurrently managed with a single treatment.

The main limitation of this study relates to its preliminary and exploratory nature, based on expert opinions, not on patient opinions, which requires further research in the clinical arena to corroborate its findings. Also, the selection of participants was not performed randomly, but after MS specialists' availability and willingness to participate and confirmation until reaching the predetermined participant number, so our sample may not be representative of the neurologist community.

This study is of relevant value because it assessed the suitability of the new concept of Spasticity-Plus syndrome in clinical practice by integrating the experience of 55 Spanish neurologists with extensive expertise in MS management. The structured discussion and sharing of clinical experience among a group of experts allowed by the Workmat^®^ methodology is critically important in the context of empirically inductive research, like the present one, aimed to build new hypotheses to be further tested in the clinical setting. The conclusions of this study can guide future investigations to achieve a deeper understanding of the integrative management of MS spasticity and associated symptoms.

## Conclusion

A panel of 55 Spanish MS specialists agreed that several symptoms within the Spasticity-Plus syndrome such as pain, spasms/cramps, bladder dysfunction, and others may appear concomitantly in MS patients with spasticity, impairing their QoL, and thus should be considered a high treatment priority. According to the specialists' clinical experience, Nabiximols treatment satisfactorily improves several concomitant symptoms beyond spasticity. The unified management of the Spasticity-Plus syndrome may open the door to optimizing symptom treatment in MS patients.

## Data Availability Statement

The raw data supporting the conclusions of this article will be made available by the authors, without undue reservation.

## Ethics Statement

Ethical review and approval was not required for the study on participants in accordance with the local legislation and institutional requirements. Participants provided their written consent for their participation in the project through the corresponding contract signature.

## Author Contributions

All authors participated in the new concept conception. All authors aided in designing and testing the exercises for discussion. OF, LC-F, MM-G, JP-G, and LR-T acted as moderators in online meetings and all authors discussed and interpreted the results. OF prepared the first draft of the manuscript and all authors contributed to manuscript revision, read, and approved the submitted version.

## Conflict of Interest

The study was funded by an investigational grant from Almirall SA. The funder had no role in study design, data collection and analysis, decision to publish, or preparation of the manuscript. Authors' views and opinions are not necessarily aligned with those of Almirall SA. OF has received honoraria as consultant in advisory boards, as chair/lecturer in meetings, and from participation in clinical trials and other research projects promoted by Actelion, Allergan, Almirall, Bayer-Schering, Biogen-Idec, Genzyme, Merck-Serono, Novartis, Orizon, Roche, and Teva. LC-F has received honoraria as consultant in advisory boards, travel support, and speaker fees and from participation in clinical trials and other research projects promoted by Almirall, Bayer, Biogen-Idec, Biopas, Bristol Myers Squibb, Merck-Serono, Novartis, Roche, Sanofi-Genzyme, and Teva. MM-G has received compensation for consulting services and speaking fees and from participation in clinical trials from Almirall, Bayer-Schering, Biogen-Idec, Bristol Myers Squibb, Merck, Novartis, Roche, Sanofi-Genzyme, and Teva. PM has received honoraria as consultant in advisory boards, travel support, and speaker fees and from participation in clinical trials and other research projects promoted by Allergan, Almirall, Biogen-Idec, Merck-Serono, Merz, Sanofi-Genzyme and, Teva. JP-G has received honoraria as consultant for Bayer Pharmaceuticals, Biogen Spain S.L., Genzyme Corporation, Merck Serono, Novartis Pharmaceuticals Corporation, Sanofi, Teva Pharmaceutical Industries, Roche Pharma, Almirall Prodesfarma S.A., and Celgene España S.L. JP-G has participated as lecturer/moderator at meetings and/or symposia organized by Almirall Prodesfarma S.A., Bayer Pharmaceuticals, Biogen Spain S.L., Genzyme Corporation, Merck Serono, Novartis Pharmaceuticals Corporation, Sanofi, Teva Pharmaceutical Industries, and Roche Pharma. JP-G has received funding for research projects from Almirall Prodesfarma S.A., Biogen Spain S.L., Novartis Pharmaceutical Corporation, Teva Pharmaceutical Industries, and Sanofi. LR-T has received honoraria as consultant in advisory boards, as chair/lecturer in meetings, and from participation in clinical trials and other research projects promoted by Almirall, Bayer, Biogen-Idec, Sanofi, Genzyme, Merck, Novartis, Roche, Bristol-Myers, and Teva.

## Publisher's Note

All claims expressed in this article are solely those of the authors and do not necessarily represent those of their affiliated organizations, or those of the publisher, the editors and the reviewers. Any product that may be evaluated in this article, or claim that may be made by its manufacturer, is not guaranteed or endorsed by the publisher.

## Spasticity-Plus Working Group

Aguera Morales, E; Aladro Benito, Y; Alonso Torres, A; Álvarez de Arcaya Esquide, A; Álvarez Rodríguez, E; Arnal García, MC; Ayuso Peralta, L; Barrero Hernández, FJ; Belenguer Benavides, A; Blanco Morgado, Y; Brieva Ruiz, L; Caminero Rodríguez, AB; Candeliere Merlicco, A; Cano Orgaz, A; Carcelén Gade, ME; Castillo Triviño, T; Costa Arpín, E; Cuello, JP; Dávila González, P; Domínguez Morán, JA; Dotor García-Soto, J; Durán Ferreras, E; Eguía del Río, P; Fernández Díaz, EM; Fernández Uría, D; Forero Diaz, L; Garcés Redondo, M; García Castañón, I; García Domínguez, JM; González Quintanilla, V; Hervàs Pujol, M; Jiménez Caballero, PE; Labiano Fontcuberta, A; Landete Pascual, L; Llaneza González, MA; Meca Lallana, V; Mestre Sansó, M; Miralles Martínez, AA; Moral Torres, E; Muñoz Fernández, MC; Otano Martínez, M; Oterino Durán, A; Peña Martínez, J; Pérez Miralles, F; Pérez Sempere, A; Pérez Vicente, JA; Ramo Tello, C; Reyes Garrido, V; Romera Tellado, M; Sainz de la Maza Cantero, S; Sebastian Torres, B; Solà Valls, N; Sotoca Fernández, JJ; Tallón Barranco, A; Villafani Echazú, J.

## References

[1] Wallin MT Culpepper WJ. Global, regional, and national burden of multiple sclerosis 1990-2016: a systematic analysis for the Global Burden of Disease Study 2016 GBD 2016 Multiple Sclerosis Collaborators*. Artic Lancet Neurol. (2019) 18:269–85. 10.1016/S1474-4422(18)30443-5 30679040 PMC6372756

[2] Coetzee T Thompson AJ. Atlas of MS 2020: Informing global policy change. Mult Scler J. (2020) 26:1807–8. 10.1177/1352458520968811 33174499

[3] Pérez-Carmona N Fernández-Jover E Sempere ÁP. Epidemiology of multiple sclerosis in Spain. Rev Neurol. (2019) 69:32–8. 10.33588/rn.6901.2018477 31236909

[4] Thompson AJ Baranzini SE Geurts J Hemmer B Ciccarelli O. Multiple sclerosis. Lancet. (2018) 391:1622–36. 10.1016/S0140-6736(18)30481-1 29576504

[5] Filippi M Bar-Or A Piehl F Preziosa P Solari A Vukusic S. Multiple sclerosis. Nat Rev Dis Prim. (2018) 4:43. 10.1038/s41572-018-0041-4 30410033

[6] Piehl F. A changing treatment landscape for multiple sclerosis: challenges and opportunities. J Intern Med. (2014) 275:364–81. 10.1111/joim.12204 24444084

[7] Filippi M Rocca MA. Rethinking multiple sclerosis treatment strategies. Lancet Neurol. (2020) 19:281–2. 10.1016/S1474-4422(20)30063-6 32199086

[8] Tintore M Vidal-Jordana A Sastre-Garriga J. Treatment of multiple sclerosis — success from bench to bedside. Nat Rev Neurol. (2019) 15:53–8. 10.1038/s41582-018-0082-z 30315270

[9] Lunde HMB Assmus J Myhr K-M Bø L Grytten N. Survival and cause of death in multiple sclerosis: a 60-year longitudinal population study. J Neurol Neurosurg Psychiatry. (2017) 88:621–5. 10.1136/jnnp-2016-315238 28365589 PMC5537547

[10] Kister I Bacon TE Chamot E Salter AR Cutter GR Kalina JT Herbert J. Natural history of multiple sclerosis symptoms. Int J MS Care. (2013) 15:146–58. 10.7224/1537-2073.2012-053 24453777 PMC3883021

[11] Fernández Ó Costa-Frossard L Martínez-Ginés M Montero P Prieto JM Ramió L. The broad concept of “spasticity-plus syndrome” in multiple sclerosis: a possible new concept in the management of multiple sclerosis symptoms. Front Neurol. (2020) 11:1–8. 10.3389/fneur.2020.00152 32256440 PMC7090019

[12] Fletcher SG Castro-Borrero W Remington G Treadaway K Lemack GE Frohman EM. Sexual dysfunction in patients with multiple sclerosis: a multidisciplinary approach to evaluation and management. Nat Clin Pract Urol. (2009) 6:96–107. 10.1038/ncpuro1298 19198623

[13] Frahm N Hecker M Zettl UK. Polypharmacy among patients with multiple sclerosis: a qualitative systematic review. Expert Opin Drug Saf. (2020) 19:139–45. 10.1080/14740338.2020.1720646 31965869

[14] Thelen JM Lynch SG Bruce AS Hancock LM Bruce JM. Polypharmacy in multiple sclerosis: Relationship with fatigue, perceived cognition, and objective cognitive performance. J Psychosom Res. (2014) 76:400–4. 10.1016/j.jpsychores.2014.02.013 24745782

[15] Arroyo R Vila C Clissold S. Retrospective observational study of the management of multiple sclerosis patients with resistant spasticity in Spain: the “5E” study. Expert Rev Pharmacoeconomics Outcomes Res. (2011) 11:205–13. 10.1586/erp.11.6 21476822

[16] Rizzo MA Hadjimichael OC Preiningerova J Vollmer TL. Prevalence and treatment of spasticity reported by multiple sclerosis patients. Mult Scler. (2004) 10:589–95. 10.1191/1352458504ms1085oa 15471378

[17] Oreja-Guevara C González-Segura D Vila C. Spasticity in multiple sclerosis: results of a patient survey. Int J Neurosci. (2013) 123:400–8. 10.3109/00207454.2012.762364 23297730

[18] Van Sickle MD. Identification and Functional Characterization of Brainstem Cannabinoid CB2 Receptors. Science (80-). (2005) 310:329–32. 10.1126/science.1115740 16224028

[19] Wade DT Makela P Robson P House H Bateman C. Do cannabis-based medicinal extracts have general or specific effects on symptoms in multiple sclerosis? A double-blind, randomized, placebo-controlled study on 160 patients. Mult Scler J. (2004) 10:434–41. 10.1191/1352458504ms1082oa 15327042

[20] Collin C Ehler E Waberzinek G Alsindi Z Davies P Powell K. A double-blind, randomized, placebo-controlled, parallel-group study of Sativex, in subjects with symptoms of spasticity due to multiple sclerosis. Neurol Res. (2010) 32:451–9. 10.1179/016164109X12590518685660 20307378

[21] Novotna A Mares J Ratcliffe S Novakova I Vachova M Zapletalova O. A randomized, double-blind, placebo-controlled, parallel- group, enriched-design study of nabiximols* (Sativex ^®^), as add-on therapy, in subjects with refractory spasticity caused by multiple sclerosis. Eur J Neurol. (2011) 18:1122–31. 10.1111/j.1468-1331.2010.03328.x 21362108

[22] Markovà J Essner U Akmaz B Marinelli M Trompke C Lentschat A. Sativex ^®^ as add-on therapy vs. further optimized first-line ANTispastics (SAVANT) in resistant multiple sclerosis spasticity: a double-blind, placebo-controlled randomized clinical trial. Int J Neurosci. (2019) 129:119–28. 10.1080/00207454.2018.1481066 29792372

[23] Patti F Chisari CG Solaro C Benedetti MD Berra E Bianco A. Effects of THC/CBD oromucosal spray on spasticity-related symptoms in people with multiple sclerosis: results from a retrospective multicenter study. Neurol Sci. (2020) 41:2905–13. 10.1007/s10072-020-04413-6 32335779

[24] Anguita-Sánchez M Marco-Vera P Alonso-Moreno FJ Arribas-Ynsaurriaga F Gállego-Culleré J Honorato-Pérez J. Percepción de los médicos sobre los factores que influyen en la elección de un dicumarínico o de un nuevo anticoagulante oral en pacientes con fibrilación auricular no valvular. Atención Primaria. (2016) 48:527–34. 10.1016/j.aprim.2015.11.004 26971361 PMC6877842

[25] Martínez-Raga J Amore M Di Sciascio G Florea RI Garriga M Gonzalez G. 1st international experts' meeting on agitation: Conclusions regarding the current and ideal management paradigm of agitation. Front Psychiatry. (2018) 9:1–9. 10.3389/fpsyt.2018.00054 29535649 PMC5835036

[26] Ward JH. Hierarchical grouping to optimize an objective function. J Am Stat Assoc. (1963) 58:236–44. 10.1080/01621459.1963.10500845

[27] Thompson AJ Toosy AT Ciccarelli O. Pharmacological management of symptoms in multiple sclerosis: current approaches and future directions. Lancet Neurol. (2010) 9:1182–99. 10.1016/S1474-4422(10)70249-0 21087742

[28] Milinis K Tennant A Young CA. Spasticity in multiple sclerosis: associations with impairments and overall quality of life. Mult Scler Relat Disord. (2016) 5:34–9. 10.1016/j.msard.2015.10.007 26856941

[29] Vivancos-Matellano F Pascual-Pascual SI Nardi-Vilardaga J Miquel-Rodríguez F De Miguel-León I Martínez-Garre MC. Guide to the comprehensive treatment of spasticity. Rev Neurol. (2007) 45:365–75. 10.33588/rn.4506.2007239 17899519

[30] Flachenecker P Henze T Zettl UK. Spasticity in patients with multiple sclerosis - clinical characteristics, treatment and quality of life. Acta Neurol Scand. (2014) 129:154–62. 10.1111/ane.12202 24256407

[31] Nortvedt MW Riise T Myhr KM Landtblom AM Bakke A Nyland HI. Reduced quality of life among multiple sclerosis patients with sexual disturbance and bladder dysfunction. Mult Scler. (2001) 7:231–5. 10.1191/135245801680209330 11548982

[32] Bakshi R. Fatigue associated with multiple sclerosis: diagnosis, impact and management. Mult Scler. (2003) 9:219–27. 10.1191/1352458503ms904oa 12814166

[33] Patti F. Cognitive impairment in multiple sclerosis. Mult Scler. (2009) 15:2–8. 10.1177/1352458508096684 18805842

[34] Motl RW McAuley E. Symptom cluster and quality of life: preliminary evidence in multiple sclerosis. J Neurosci Nurs. (2010) 42:212–6. 10.1097/JNN.0b013e3181e26c5f 20804116 PMC2956182

[35] Motl RW Suh Y Weikert M. Symptom cluster and quality of life in multiple sclerosis. J Pain Symptom Manage. (2010) 39:1025–32. 10.1016/j.jpainsymman.2009.11.312 20434872

[36] Rommer PS Eichstädt K Ellenberger D Flachenecker P Friede T Haas J. Symptomatology and symptomatic treatment in multiple sclerosis: results from a nationwide MS registry. Mult Scler J. (2019) 25:1641–52. 10.1177/1352458518799580 30230952

[37] Motl RW McAuley E. Symptom cluster as a predictor of physical activity in multiple sclerosis: preliminary evidence. J Pain Symptom Manage. (2009) 38:270–80. 10.1016/j.jpainsymman.2008.08.004 19329276

[38] Williams AE Vietri JT Isherwood G Flor A. Symptoms and association with health outcomes in relapsing-remitting multiple sclerosis: results of a US patient survey. Mult Scler Int. (2014) 2014:1–8. 10.1155/2014/203183 25328704 PMC4189937

[39] Amtmann D Askew RL Kim J Chung H Ehde DM Bombardier CH. Pain affects depression through anxiety, fatigue, and sleep in multiple sclerosis. Rehabil Psychol. (2015) 60:81–90. 10.1037/rep0000027 25602361 PMC4349204

[40] Coyle PK. Symptom management and lifestyle modifications in multiple sclerosis. Contin Lifelong Learn Neurol. (2016) 22:815–36. 10.1212/CON.0000000000000325 27261684

[41] Workman CD Kamholz J Rudroff T. Transcranial direct current stimulation (tDCS) for the treatment of a Multiple Sclerosis symptom cluster. Brain Stimul. (2020) 13:263–4. 10.1016/j.brs.2019.09.012 31585722

